# Crystal structure, Hirshfeld surface analysis and anti­oxidant capacity of 2,2′-{(1*E*,1′*E*)-[1,2-phenyl­enebis(aza­nylyl­idene)]bis­(methanylyl­idene)}bis­(5-benz­yloxy)phenol

**DOI:** 10.1107/S2056989018005832

**Published:** 2018-04-19

**Authors:** Nadir Ghichi, Ali Benboudiaf, Yacine DJebli, Chawki Bensouici, Hocine Merazig

**Affiliations:** a Unit of Research CHEMS, University of Constantine1, Algeria; b Laboratory of Materials Chemistry, University of Constantine1, Algeria; c Biotechnology Research Center, Constantine, Algeria

**Keywords:** crystal structure, Schiff base, anti­oxidant capacity, CUPRAC, hydrogen bonding, C—H⋯π inter­actions, Hirshfeld surface analysis

## Abstract

The whole mol­ecule of the title Schiff base compound, C_34_H_28_N_2_O_4_, is generated by mirror symmetry, with the mirror bis­ecting the central benzene ring. It was synthesized *via* the condensation reaction of 1,2-di­amine­benzene with 4-benz­yloxy-2-hy­droxy­benzaldehyde. The mol­ecule is V-shaped and there are two intra­molecular O—H⋯N hydrogen bonds present forming *S*(6) ring motifs. The configuration about the C=N imine bonds is *E*. The central benzene ring makes dihedral angles of 41.9 (2) and 43.6 (2)° with the phenol ring and the outer benz­yloxy ring, respectively. The latter two rings are inclined to each other by 84.4 (2)°. In the crystal, mol­ecules are linked by C—H⋯π inter­actions, forming layers lying parallel to the *ab* plane. The Hirshfeld surface analysis and the two-dimensional fingerprint plots confirm the predominance of these inter­actions in the crystal structure. The anti­oxidant capacity of the compound was determined by the cupric reducing anti­oxidant capacity (CUPRAC) process.

## Abstract 2

The title Schiff base compound was synthesized *via* the condensation reaction of 1,2-di­amine­benzene with 4-benz­yloxy-2-hy­droxy­benzaldehyde. The mol­ecule is V-shaped and possesses mirror symmetry; the mirror bis­ects the central benzene ring. There are two intra­molecular O—H⋯N hydrogen bonds present forming *S*(6) ring motifs.

## Chemical context   

Schiff base derivatives are a biologically versatile class of compounds possessing diverse activities, such as anti-oxidant (Haribabu *et al.*, 2015 ▸, 2016 ▸), anti-inflammatory (Alam *et al.*, 2012 ▸), anti­anxiety, anti­depressant (Jubie *et al.*, 2011 ▸), anti-tumour, anti­bacterial, and fungicidal properties (Refat *et al.*, 2008 ▸; Kannan & Ramesh, 2006 ▸). Bis-bidentate Schiff base ligands have been studied extensively and used as building blocks in metallo-supra­molecular chemistry (Birkedal & Pattison, 2006 ▸; Shahverdizadeh & Tiekink, 2011 ▸; Chu & Huang, 2007 ▸; Yoshida & Ichikawa, 1997 ▸; Kruger *et al.*, 2001 ▸). The common structural feature of these compounds is the presence of an azomethine group, linked by a *η* methyl­ene bridge, which can act as a hydrogen-bond acceptor. In view of this inter­est we have synthesized the title compound, (I), and report herein on its crystal structure. The ^1^H NMR NMR spectrum reveals the presence of an imino group (N=CH) in the range δ = 8.5–8.7 p.p.m. The anti­oxidant capacity of the compound was determined by the cupric reducing anti­oxidant capacity (CUPRAC) process.
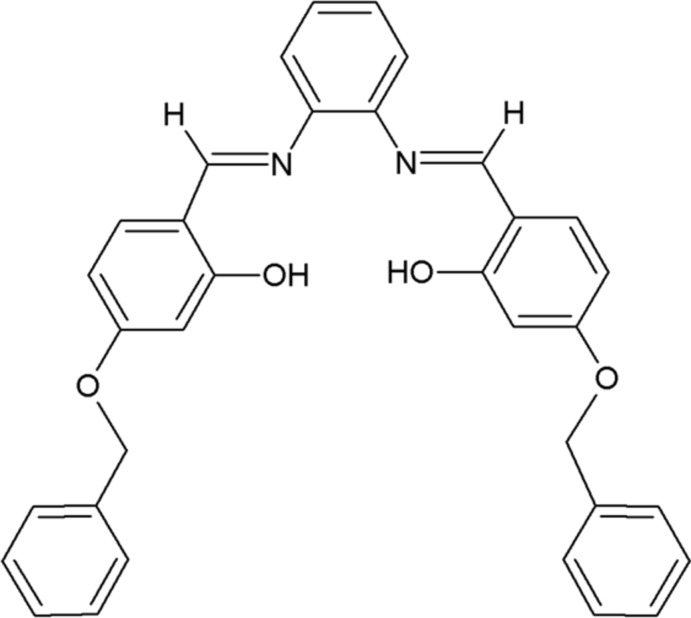


## Structural commentary   

**Figure 1:**
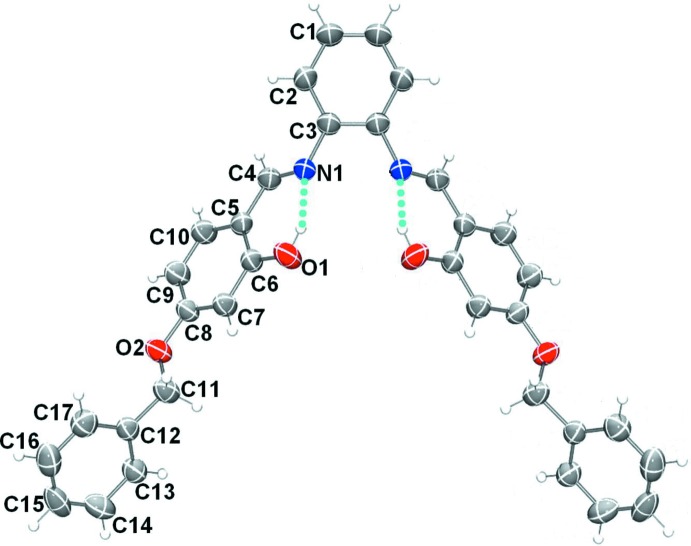
View of the mol­ecular structure of compound (I), with atom labelling. Displacement ellipsoids are drawn at the 50% probability level. Unlabelled atoms are related to labelled atoms by the mirror symmetry code: (i) −*x*, *y*, *z*. The intra­molecular O—H⋯N hydrogen bonds (see Table 1 ▸) are shown as dashed lines.

The mol­ecular structure of compound (I) is illustrated in Fig. 1 ▸. The asymmetric unit consists of half a mol­ecule, with the whole mol­ecule being generated by mirror symmetry. The mirror bis­ects the central benzene ring, *viz.* bonds C1—C1^i^ and C3—C3^i^ [symmetry code: (i) −*x*, *y*, *z*]. In the mol­ecule there are two intra­molecular O—H⋯N hydrogen bonds present (Table 1 ▸), which form *S*(6) ring motifs as shown in Fig. 1 ▸. The configuration of the C4=N1 imine bonds is *E* and the C4=N1 bond length is 1.278 (6) Å. The C3—N1=C4 bond angles are less than 120° [118.9 (4)°], and the imine group has a C3—N1—C4—C5 torsion angle of −176.8 (4)°. The mol­ecule is V-shaped and the two arms are non-planar; the central benzene ring forms dihedral angles of 41.9 (2) and 43.6 (2)° with the phenol ring (C5-C10) and the outer benz­yloxy ring (C12–C17), respectively. The latter two rings are almost normal to each other, with a dihedral angle of 84.4 (2)°.

## Supra­molecular features and Hirshfeld surface analysis   

**Figure 2:**
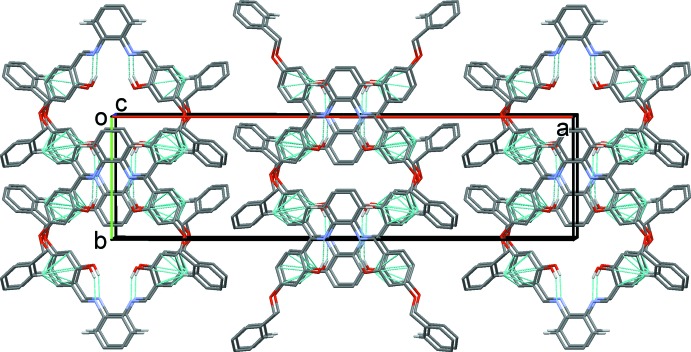
Crystal packing of compound (I) viewed along the *c* axis, with the O—H⋯N intra­molecular hydrogen bonds and the C—H⋯π inter­actions (see Table 1 ▸) illustrated as dashed lines.

In the crystal of (I), mol­ecules are linked by C—H⋯π inter­actions (Table 1 ▸), forming layers parallel to the (001) plane, as illustrated in Fig. 2 ▸.

**Figure 3:**
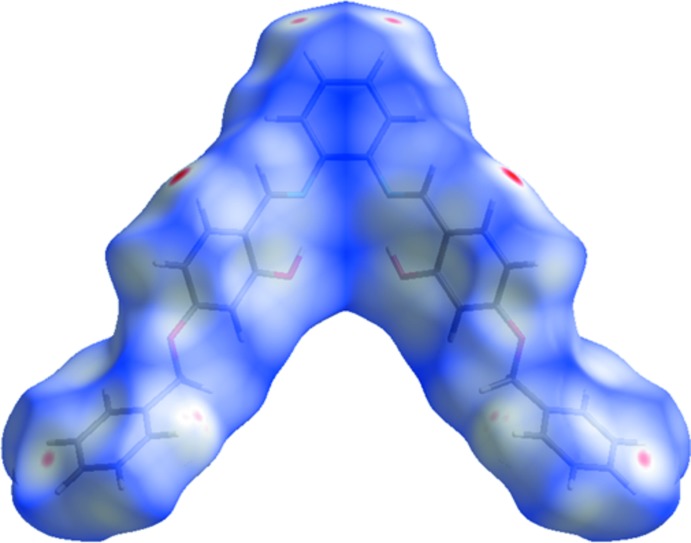
View of the Hirshfeld surface of (I) mapped over *d*_norm_.

**Figure 4:**
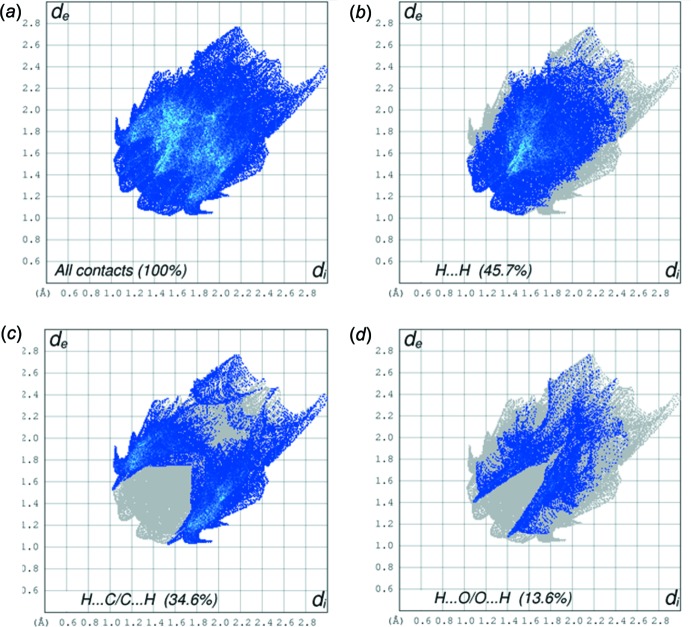
The two-dimensional fingerprint plots of (I): (*a*) all inter­actions; (*b*) H⋯H; (*c*) H⋯C/C⋯H; (*d*) H⋯O/O⋯H.

The Hirshfeld surface analysis (Spackman & Jayatilaka, 2009 ▸) and the associated two-dimensional fingerprint plots (McKinnon *et al.*, 2007 ▸) were performed with *CrystalExplorer17* (Turner *et al.*, 2017 ▸). The Hirshfeld surface of compound (I) mapped over *d*_norm_ is given in Fig. 3 ▸, and the fingerprint plots are given in Fig. 4 ▸. They reveal that the principal inter­molecular inter­actions are H⋯H at 45.7% (Fig. 4 ▸ *b*) and H⋯C/C⋯H at 34.6% (Fig. 4 ▸ *c*), followed by the H⋯O/O⋯H inter­actions at 13.6% (Fig. 4 ▸ *d*).

## Database survey   

**Figure 5:**
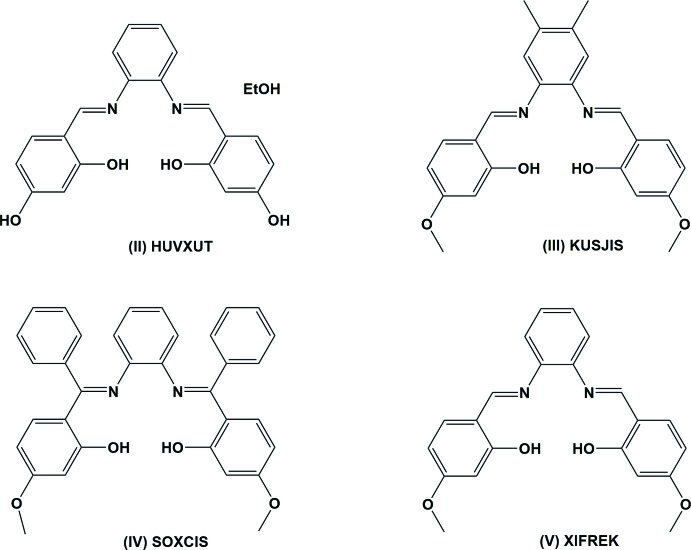
Similar compounds to that of the title compound, (I), in the CSD; see Section 4, *Database survey*.

A search of the Cambridge Structural Database (CSD, Version 5.39, last update February 2018; Groom *et al.*, 2016 ▸) for similar compounds yielded four hits. These compounds (see Fig. 5 ▸) include 5,5′-dihy­droxy-2,2′-[*o*-phenyl­enebis(nitrilo­methyl­ene)]diphenol ethanol solvate (II) (CSD refcode HUVXUT; Soroceanu *et al.*, 2013 ▸), 5,5′-dimeth­oxy-2,2′-[4,5-dimethyl-*o*-phenyl­enebis(nitrilo­methyl­idyne)]diphenol (III) (KUSJIS; Kargar *et al.*, 2010 ▸), 1,2-bis­{[(2-hy­droxy-4-meth­oxy­phen­yl)(phen­yl)methyl­ene]amino}­benzene (IV) (SOXCIS; Lippe *et al.*, 2009 ▸) and 5,5′-dimeth­oxy-2,2′-1,2-phenyl­enebis(nitrilo­methyl­idyne)]diphenol (V) (XIFREK; Eltayeb *et al.*, 2007 ▸). In all four compounds there are two intra­molecular O—H⋯N hydrogen bonds present forming *S*(6) ring motifs.

In (II) the phenol rings are inclined to the central benzene ring by 53.9 (3) and 4.0 (2)° and to each other by 49.9 (2)°. In (III) the corresponding dihedral angles are 48.12 (8), 21.44 (8) and 47.70 (8)°, while in (V) the corresponding dihedral angles are 58.29 (12), 2.20 (12) and 57.60 (12)°. In compound (IV), that possesses twofold rotational symmetry with the twofold axis bis­ecting the central benzene ring, the phenol rings are inclined to the central benzene ring by 82.30 (5)° and to each other by 63.76 (5)°. In the title compound, which possesses mirror symmetry, the corresponding dihedral angles are 41.9 (2) and 68.9 (2)°.

A search of the CSD for metal complexes of compounds similar to compound (I) gave over 30 hits. The ligands always coordinate in a tetra­dentate manner. For example, there were 13 hits for transition metal complexes of compound (II). The majority involve square-planar coordinated metal atoms, such as in complexes (5,5′-dihy­droxy-2,2′-[*o*-phenyl­enebis(nitrilo­methyl­idyne)]diphenolato)nickel(II) dihydrate (POFFOG; Fun *et al.*, 2008 ▸) and (4,4′-{1,2-phenyl­enebis[(nitrilo-κ*N*)methylyl­idene]}di­benzene-1,3-diolato-κ*O* ^3^)copper(II) methanol solvate (DUQBEX; Niu *et al.*, 2010 ▸). For compound (V), five hits were found; they include three sixfold-coord­inated tin complexes (DOSCOF, DOSDAS, DOSFOI; Muñoz-Flores *et al.*, 2014 ▸) and two square-pyramidal manganese complexes (ODESEY, Ghaemi *et al.*, 2016 ▸; XIYQOM, Eltayeb *et al.*, 2008 ▸).

## Anti­oxidant activity   

**Figure 6:**
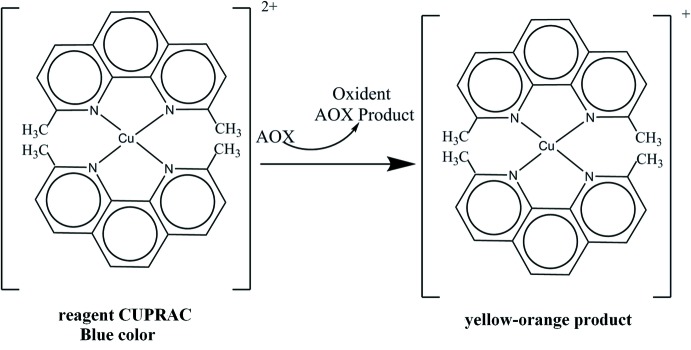
Reduction of the chromogenic complex of Cu^+2^–Nc

The anti­oxidant activity profile of the synthesized compound (I) was determined by utilizing the copper(II)–neocuprine [Cu^II^-Nc] (CUPRAC) method (Apak *et al.*, 2004 ▸). The CUPRAC method (Fig. 6 ▸) (cupric ion reducing anti­oxidant capacity) is based on the follow-up of the decrease in the increased absorbance of the neocuproene (Nc), copper (Cu^+2^)Nc_2_–Cu^+2^ complex. Indeed, in the presence of an anti­oxidant agent, the copper–neocuproene complex is reduced and this reaction is qu­anti­fied spectrophotometrically at a wavelength of 450 nm.

According to the cupric ion reducing anti­oxidant capacity assay, the title compound displayed activity with variable potency in all tested concentrations, because the percentage (%) inhibition in the CUPRAC assay is good [A_0.50_ = 15.03 ± 1.50 for a 4 mg dosage, compared to the results for buthylated toluene (BHT) [A_0.50_ = 8.97 ± 3.94], used as a positive control (see Table 2 ▸). Note: In CUPRAC anti­oxidant activity, the values expressed are the mean ± s.u.s of three parallel measurements (*p* < 0.05).

## Synthesis and crystallization   

1,2-Di­amine­benzene (0.027 g) and 4-benz­yloxy-2-hy­droxy­benzaldehyde (0.1141 g) in ethanol (15 ml) were refluxed for 1 h, then the solvent was evaporated *in vacuo*. The residue was recrystallized from ethanol, yielding yellow block-like crystals of the title compound on slow evaporation of the solvent. The purity of the compound was characterized by its NMR spectrum (250 MHz, CDCl_3_). The azomethine proton appears in the 8.5–8.7 p.p.m. range, while the imine bond is characterized in the ^13^C RMN spectrum with the imine C and OH signals in the range 162.23–163.34 p.p.m. ^1^H NMR: δ = 6.5–7.6 (*m*, 12H; *H-ar*), δ = 13.7 (*s*, 1H; *OH*), δ = 5.1 (*s*, 1H; *CH_2_*–O). ^13^C NMR: 70.15, 120.33, 127.30, 127.64, 128.26, 128.75, 142.32, 162.23, 163.33, 163.34.

## Refinement   

Crystal data, data collection and structure refinement details are summarized in Table 3 ▸. The hydroxyl H atom was located in a difference-Fourier map and initially freely refined. In the final cycles of refinements it was positioned geometrically (O—H = 0.82 Å) and refined as riding with *U*_iso_(H) = 1.5*U*_eq_(O). The C-bound H atoms were positioned geometrically (C–H = 0.93–0.97 Å) and refined as riding with *U*_iso_(H) = 1.2*U*_eq_(C).

## Supplementary Material

Crystal structure: contains datablock(s) Global, I. DOI: 10.1107/S2056989018005832/su5438sup1.cif

Structure factors: contains datablock(s) I. DOI: 10.1107/S2056989018005832/su5438Isup2.hkl

Supporting information file. DOI: 10.1107/S2056989018005832/su5438Isup3.cml

CCDC reference: 1837095

Additional supporting information:  crystallographic information; 3D view; checkCIF report

## Figures and Tables

**Table 1:** Hydrogen-bond geometry (Å, °) *Cg*2 is the centroid of the C5–C10 phenol ring.

*D*—H⋯*A*	*D*—H	H⋯*A*	*D*⋯*A*	*D*—H⋯*A*
O1—H1*O*⋯N1	0.82	1.90	2.622 (5)	147
C2—H2⋯*Cg*2^i^	0.93	2.88	3.499 (5)	125
C13—H13⋯*Cg*2^ii^	0.93	2.60	3.493 (5)	161

Symmetry codes: (i) 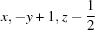; (ii) 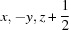.

**Table 2:** Cupric ion reducing anti­oxidant capacity of compound (I)

	Percentage (%) Inhibition							
	12.5 µg	25 µg	50 µg	100 µg	200 µg	400 µg	800 µg	A0.50 (μg ml^−1^)
Compound (I)	0.39±0.01	0.59±0.01	0.91±0.03	1.42±0.02	1.84±0.36	3.12±0.25	4.29±0.11	15.03±1.50
BHT	1.41±0.03	2.22±0.05	2.42±0.02	2.50±0.01	2.56±0.05	2.86±0.07	3.38±0.13	8.97±3.94

**Table 3:** Experimental details

Crystal data	
Chemical formula	C_34_H_28_N_2_O_4_
*M*_r_	528.58
Crystal system, space group	Orthorhombic, *C* *m* *c*2_1_
Temperature (K)	293
*a*, *b*, *c* (Å)	35.297 (3), 9.3902 (6), 8.3603 (5)
*V* (Å^3^)	2771.0 (3)
*Z*	4
Radiation type	Mo *K*α
μ (mm^−1^)	0.08
Crystal size (mm)	0.03 × 0.02 × 0.01

Data collection	
Diffractometer	Bruker APEXII CCD
No. of measured, independent and observed [*I* > 2σ(*I*)] reflections	4493, 2516, 1691
*R*_int_	0.042
(sin θ/λ)_max_ (Å^−1^)	0.651

Refinement	
*R*[*F* ^2^ > 2σ(*F* ^2^)], *wR*(*F* ^2^), *S*	0.053, 0.158, 1.02
No. of reflections	2516
No. of parameters	185
No. of restraints	1
H-atom treatment	H-atom parameters constrained
Δρ_max_, Δρ_min_ (e Å^−3^)	0.29, −0.24

Computer programs: *APEX2* and *SAINT* (Bruker, 2011 ▸), *SHELXT2017* (Sheldrick, 2015*a* ▸), *SHELXL2017* (Sheldrick, 2015*b* ▸), *SHELXTL* (Sheldrick, 2008 ▸), *Mercury* (Macrae *et al.*2008 ▸) and *PLATON* (Spek, 2009 ▸).

## supplementary crystallographic information

### Crystal data

**Table:**

C_34_H_28_N_2_O_4_	*F*(000) = 1112
*M**_r_* = 528.58	*D*_x_ = 1.267 Mg m^−^^3^
Orthorhombic, *C**m**c*2_1_	Mo *K*α radiation, λ = 0.71073 Å
Hall symbol: C 2c -2	Cell parameters from 1621 reflections
*a* = 35.297 (3) Å	θ = 2.2–21.3°
*b* = 9.3902 (6) Å	µ = 0.08 mm^−^^1^
*c* = 8.3603 (5) Å	*T* = 293 K
*V* = 2771.0 (3) Å^3^	Block, yellow
*Z* = 4	0.03 × 0.02 × 0.01 mm

### Data collection

**Table:**

Bruker APEXII CCD diffractometer	*R*_int_ = 0.042
Detector resolution: 18.4 pixels mm^-1^	θ_max_ = 27.5°, θ_min_ = 3.7°
φ and ω scans	*h* = −45→40
4493 measured reflections	*k* = −12→5
2516 independent reflections	*l* = −10→6
1691 reflections with *I* > 2σ(*I*)	

### Refinement

**Table:**

Refinement on *F*^2^	Primary atom site location: structure-invariant direct methods
Least-squares matrix: full	Secondary atom site location: difference Fourier map
*R*[*F*^2^ > 2σ(*F*^2^)] = 0.053	Hydrogen site location: mixed
*wR*(*F*^2^) = 0.158	H-atom parameters constrained
*S* = 1.01	*w* = 1/[σ^2^(*F*_o_^2^) + (0.0817*P*)^2^] where *P* = (*F*_o_^2^ + 2*F*_c_^2^)/3
2516 reflections	(Δ/σ)_max_ < 0.001
185 parameters	Δρ_max_ = 0.29 e Å^−^^3^
1 restraint	Δρ_min_ = −0.24 e Å^−^^3^

### Special details

**Table:**

Geometry. Bond distances, angles etc. have been calculated using the rounded fractional coordinates. All su's are estimated from the variances of the (full) variance-covariance matrix. The cell esds are taken into account in the estimation of distances, angles and torsion angles

### Fractional atomic coordinates and isotropic or equivalent isotropic displacement parameters (Å^2^)

**Table:**

	*x*	*y*	*z*	*U*_iso_*/*U*_eq_	
O1	0.05052 (9)	0.2350 (3)	0.3234 (5)	0.0693 (13)	
O2	0.15676 (8)	0.0288 (3)	0.5919 (4)	0.0546 (9)	
N1	0.03881 (9)	0.5084 (4)	0.3640 (5)	0.0487 (10)	
C1	0.01955 (13)	0.8879 (4)	0.2654 (7)	0.0639 (15)	
C2	0.03890 (12)	0.7645 (4)	0.2985 (6)	0.0567 (14)	
C3	0.01982 (11)	0.6379 (4)	0.3322 (5)	0.0481 (11)	
C4	0.06841 (12)	0.5103 (4)	0.4519 (6)	0.0495 (14)	
C5	0.09057 (11)	0.3828 (4)	0.4833 (5)	0.0454 (11)	
C6	0.08042 (11)	0.2506 (4)	0.4207 (5)	0.0475 (11)	
C7	0.10168 (11)	0.1285 (4)	0.4566 (5)	0.0488 (11)	
C8	0.13349 (11)	0.1401 (4)	0.5523 (5)	0.0463 (12)	
C9	0.14420 (12)	0.2710 (4)	0.6146 (6)	0.0560 (16)	
C10	0.12269 (12)	0.3889 (4)	0.5813 (5)	0.0560 (16)	
C11	0.14781 (12)	−0.1086 (4)	0.5260 (6)	0.0547 (16)	
C12	0.17886 (11)	−0.2104 (4)	0.5695 (5)	0.0449 (11)	
C13	0.17544 (12)	−0.2993 (4)	0.6998 (6)	0.0543 (16)	
C14	0.20330 (16)	−0.3988 (4)	0.7345 (6)	0.0677 (17)	
C15	0.23502 (14)	−0.4070 (5)	0.6404 (7)	0.0700 (19)	
C16	0.23920 (14)	−0.3165 (6)	0.5124 (7)	0.0697 (17)	
C17	0.21118 (13)	−0.2190 (5)	0.4765 (6)	0.0617 (17)	
H1	0.03276	0.97121	0.24301	0.0770*	
H1O	0.04038	0.31254	0.30933	0.1040*	
H2	0.06524	0.76529	0.29851	0.0680*	
H4	0.0777 (10)	0.604 (4)	0.509 (5)	0.041 (9)*	
H7	0.09442	0.04030	0.41635	0.0580*	
H9	0.16571	0.27872	0.67824	0.0670*	
H10	0.12968	0.47594	0.62529	0.0670*	
H11A	0.12385	−0.14214	0.56862	0.0660*	
H11B	0.14557	−0.10203	0.41061	0.0660*	
H13	0.15420	−0.29259	0.76516	0.0650*	
H14	0.20047	−0.45974	0.82141	0.0810*	
H15	0.25371	−0.47378	0.66311	0.0840*	
H16	0.26095	−0.32099	0.44979	0.0840*	
H17	0.21407	−0.15860	0.38915	0.0740*	

### Atomic displacement parameters (Å^2^)

**Table:**

	*U*^11^	*U*^22^	*U*^33^	*U*^12^	*U*^13^	*U*^23^
O1	0.0640 (19)	0.0400 (16)	0.104 (3)	0.0056 (14)	−0.0359 (19)	−0.0063 (17)
O2	0.0595 (17)	0.0386 (14)	0.0658 (17)	0.0080 (12)	−0.0166 (15)	−0.0028 (14)
N1	0.0416 (17)	0.0335 (16)	0.071 (2)	0.0021 (14)	0.0019 (18)	0.0024 (15)
C1	0.063 (3)	0.0317 (19)	0.097 (3)	−0.0042 (16)	−0.001 (3)	0.003 (2)
C2	0.047 (2)	0.041 (2)	0.082 (3)	−0.0048 (17)	−0.002 (2)	0.000 (2)
C3	0.049 (2)	0.0322 (18)	0.063 (2)	0.0022 (16)	0.002 (2)	−0.0028 (17)
C4	0.049 (2)	0.0345 (19)	0.065 (3)	0.0002 (17)	0.003 (2)	−0.0024 (18)
C5	0.042 (2)	0.0373 (19)	0.057 (2)	−0.0015 (16)	0.002 (2)	−0.0033 (18)
C6	0.042 (2)	0.0364 (19)	0.064 (2)	−0.0017 (16)	−0.008 (2)	−0.0015 (17)
C7	0.051 (2)	0.0365 (18)	0.059 (2)	−0.0007 (16)	−0.010 (2)	−0.0041 (19)
C8	0.047 (2)	0.039 (2)	0.053 (2)	0.0063 (16)	−0.0047 (19)	0.0005 (17)
C9	0.058 (3)	0.045 (2)	0.065 (3)	−0.0010 (18)	−0.017 (2)	−0.003 (2)
C10	0.060 (3)	0.039 (2)	0.069 (3)	−0.0035 (18)	−0.011 (2)	−0.006 (2)
C11	0.059 (3)	0.041 (2)	0.064 (3)	0.0043 (19)	−0.013 (2)	−0.0047 (19)
C12	0.043 (2)	0.0378 (19)	0.054 (2)	0.0001 (16)	−0.0069 (18)	−0.0056 (18)
C13	0.054 (3)	0.049 (2)	0.060 (3)	0.0042 (18)	−0.004 (2)	−0.001 (2)
C14	0.082 (3)	0.051 (3)	0.070 (3)	0.013 (2)	−0.021 (3)	0.005 (2)
C15	0.060 (3)	0.055 (3)	0.095 (4)	0.020 (2)	−0.023 (3)	−0.028 (3)
C16	0.053 (3)	0.075 (3)	0.081 (3)	0.006 (2)	0.001 (3)	−0.021 (3)
C17	0.062 (3)	0.057 (3)	0.066 (3)	−0.005 (2)	−0.002 (2)	−0.001 (2)

### Geometric parameters (Å, º)

**Table:**

O1—C6	1.341 (5)	C12—C13	1.378 (6)
O2—C8	1.370 (5)	C13—C14	1.387 (6)
O2—C11	1.438 (5)	C14—C15	1.371 (8)
N1—C3	1.414 (5)	C15—C16	1.374 (8)
N1—C4	1.278 (6)	C16—C17	1.381 (7)
O1—H1O	0.8200	C1—H1	0.9300
C1—C1^i^	1.380 (6)	C2—H2	0.9300
C1—C2	1.373 (6)	C4—H4	1.05 (4)
C2—C3	1.395 (5)	C7—H7	0.9300
C3—C3^i^	1.399 (5)	C9—H9	0.9300
C4—C5	1.454 (5)	C10—H10	0.9300
C5—C10	1.400 (6)	C11—H11A	0.9700
C5—C6	1.394 (5)	C11—H11B	0.9700
C6—C7	1.403 (5)	C13—H13	0.9300
C7—C8	1.383 (6)	C14—H14	0.9300
C8—C9	1.388 (6)	C15—H15	0.9300
C9—C10	1.371 (6)	C16—H16	0.9300
C11—C12	1.499 (6)	C17—H17	0.9300
C12—C17	1.383 (6)		
			
C8—O2—C11	117.4 (3)	C12—C17—C16	120.5 (5)
C3—N1—C4	118.9 (4)	C2—C1—H1	120.00
C6—O1—H1O	109.00	C1^i^—C1—H1	120.00
C1^i^—C1—C2	119.8 (4)	C1—C2—H2	119.00
C1—C2—C3	121.3 (4)	C3—C2—H2	119.00
N1—C3—C3^i^	118.3 (3)	N1—C4—H4	122 (2)
C2—C3—C3^i^	118.9 (4)	C5—C4—H4	116 (2)
N1—C3—C2	122.8 (4)	C6—C7—H7	120.00
N1—C4—C5	122.2 (4)	C8—C7—H7	120.00
C4—C5—C10	120.5 (4)	C8—C9—H9	120.00
C6—C5—C10	117.7 (3)	C10—C9—H9	120.00
C4—C5—C6	121.8 (4)	C5—C10—H10	119.00
O1—C6—C7	117.5 (3)	C9—C10—H10	119.00
C5—C6—C7	120.7 (4)	O2—C11—H11A	110.00
O1—C6—C5	121.8 (3)	O2—C11—H11B	110.00
C6—C7—C8	119.6 (4)	C12—C11—H11A	110.00
O2—C8—C9	115.0 (4)	C12—C11—H11B	110.00
C7—C8—C9	120.5 (4)	H11A—C11—H11B	108.00
O2—C8—C7	124.5 (3)	C12—C13—H13	120.00
C8—C9—C10	119.2 (4)	C14—C13—H13	120.00
C5—C10—C9	122.3 (4)	C13—C14—H14	120.00
O2—C11—C12	108.6 (3)	C15—C14—H14	120.00
C11—C12—C13	120.9 (4)	C14—C15—H15	120.00
C13—C12—C17	118.8 (4)	C16—C15—H15	120.00
C11—C12—C17	120.3 (4)	C15—C16—H16	120.00
C12—C13—C14	120.8 (4)	C17—C16—H16	120.00
C13—C14—C15	119.8 (4)	C12—C17—H17	120.00
C14—C15—C16	120.0 (5)	C16—C17—H17	120.00
C15—C16—C17	120.2 (5)		
			
C11—O2—C8—C7	1.5 (6)	C4—C5—C10—C9	179.6 (4)
C11—O2—C8—C9	−177.8 (4)	C6—C5—C10—C9	0.9 (6)
C8—O2—C11—C12	174.0 (3)	O1—C6—C7—C8	177.9 (4)
C4—N1—C3—C2	41.3 (6)	C5—C6—C7—C8	−1.4 (6)
C4—N1—C3—C3^i^	−139.8 (4)	C6—C7—C8—O2	−178.4 (4)
C3—N1—C4—C5	−176.8 (4)	C6—C7—C8—C9	0.9 (6)
C1^i^—C1—C2—C3	0.1 (8)	O2—C8—C9—C10	179.9 (4)
C2—C1—C1^i^—C2^i^	0.0 (9)	C7—C8—C9—C10	0.5 (7)
C1—C2—C3—N1	178.9 (5)	C8—C9—C10—C5	−1.4 (7)
C1—C2—C3—C3^i^	−0.1 (7)	O2—C11—C12—C13	96.8 (4)
N1—C3—C3^i^—N1^i^	0.0 (6)	O2—C11—C12—C17	−84.8 (5)
N1—C3—C3^i^—C2^i^	−179.0 (4)	C11—C12—C13—C14	176.4 (4)
C2—C3—C3^i^—N1^i^	179.0 (4)	C17—C12—C13—C14	−2.0 (6)
C2—C3—C3^i^—C2^i^	0.0 (6)	C11—C12—C17—C16	−177.4 (4)
N1—C4—C5—C6	−0.9 (7)	C13—C12—C17—C16	1.0 (7)
N1—C4—C5—C10	−179.5 (4)	C12—C13—C14—C15	1.4 (7)
C4—C5—C6—O1	2.6 (6)	C13—C14—C15—C16	0.2 (7)
C4—C5—C6—C7	−178.2 (4)	C14—C15—C16—C17	−1.2 (8)
C10—C5—C6—O1	−178.8 (4)	C15—C16—C17—C12	0.6 (8)
C10—C5—C6—C7	0.5 (6)		

Symmetry code: (i) −*x*, *y*, *z*.

### Hydrogen-bond geometry (Å, º)

Cg2 is the centroid of the C5–C10 phenol ring.

**Table:**

*D*—H···*A*	*D*—H	H···*A*	*D*···*A*	*D*—H···*A*
O1—H1*O*···N1	0.82	1.90	2.622 (5)	147
C2—H2···*Cg*2^ii^	0.93	2.88	3.499 (5)	125
C13—H13···*Cg*2^iii^	0.93	2.60	3.493 (5)	161

Symmetry codes: (ii) *x*, −*y*+1, *z*−1/2; (iii) *x*, −*y*, *z*+1/2.

## References

[bb1] Alam, M. S., Choi, J.-H. & Lee, D.-U. (2012). *Bioorg. Med. Chem.* **20**, 4103–4108. 10.1016/j.bmc.2012.04.058 22626550

[bb2] Apak, R., Güçlü, K., Özyürek, M. & Karademir, S. E. (2004). *J. Agric. Food Chem.* **52**, 7970–7981. 10.1021/jf048741x 15612784

[bb3] Birkedal, H. & Pattison, P. (2006). *Acta Cryst.* C**62**, o139–o141. 10.1107/S0108270106003283 16518050

[bb4] Bruker (2011). *APEX2* and *SAINT*. Bruker AXS Inc., Madison, Wisconsion, USA.

[bb5] Chu, Z. & Huang, W. (2007). *J. Mol. Struct.* **837**, 15–22.

[bb6] Eltayeb, N. E., Teoh, S. G., Chantrapromma, S., Fun, H.-K. & Adnan, R. (2008). *Acta Cryst.* E**64**, m670–m671. 10.1107/S1600536808009835 PMC2961208 21202211

[bb7] Eltayeb, N. E., Teoh, S. G., Chantrapromma, S., Fun, H.-K. & Ibrahim, K. (2007). *Acta Cryst.* E**63**, o3094–o3095.

[bb8] Fun, H.-K., Kia, R., Mirkhani, V. & Zargoshi, H. (2008). *Acta Cryst.* E**64**, m1181–m1182. 10.1107/S1600536808026093 PMC2960688 21201624

[bb9] Ghaemi, A., Keyvani, B., Rayati, S., Zarei, S. & Notash, B. (2016). *Zh. Strukt. Khim. (Russ. J. Struct. Chem.)*, **57**, 1027–1030.

[bb10] Groom, C. R., Bruno, I. J., Lightfoot, M. P. & Ward, S. C. (2016). *Acta Cryst.* B**72**, 171–179. 10.1107/S2052520616003954 PMC4822653 27048719

[bb11] Haribabu, J., Subhashree, G. R., Saranya, S., Gomathi, K., Karvembu, R. & Gayathri, D. (2015). *J. Mol. Struct.* **1094**, 281–291.

[bb12] Haribabu, J., Subhashree, G. R., Saranya, S., Gomathi, K., Karvembu, R. & Gayathri, D. (2016). *J. Mol. Struct.* **1110**, 185–195.

[bb13] Jubie, S., Sikdar, P., Antony, S., Kalirajan, R., Gowramma, B., Gomathy, S. & Elango, K. (2011). *Pak. J. Pharm. Sci.* **24**, 109–112. 21454157

[bb14] Kannan, M. & Ramesh, R. (2006). *Polyhedron*, **25**, 3095–3103.

[bb15] Kargar, H., Kia, R., Khan, I. U., Sahraei, A. & Aberoomand Azar, P. (2010). *Acta Cryst.* E**66**, o728. 10.1107/S1600536810007282 PMC2983852 21580575

[bb16] Kruger, P. E., Martin, N. & Nieuwenhuyzen, M. (2001). *J. Chem. Soc. Dalton Trans.* pp. 1966–1970.

[bb17] Lippe, K., Gerlach, D., Kroke, E. & Wagler, J. (2009). *Organometallics*, **28**, 621–629.

[bb18] Macrae, C. F., Bruno, I. J., Chisholm, J. A., Edgington, P. R., McCabe, P., Pidcock, E., Rodriguez-Monge, L., Taylor, R., van de Streek, J. & Wood, P. A. (2008). *J. Appl. Cryst.* **41**, 466–470.

[bb19] McKinnon, J. J., Jayatilaka, D. & Spackman, M. A. (2007). *Chem. Commun.* pp. 3814–3816. 10.1039/b704980c 18217656

[bb20] Muñoz-Flores, B. M., Santillán, R., Farfán, N., Álvarez-Venicio, V., Jiménez-Pérez, V. M., Rodríguez, M., Morales-Saavedra, O. G., Lacroix, P. G., Lepetit, C. & Nakatani, K. (2014). *J. Organomet. Chem.* **769**, 64–71.

[bb21] Niu, M., Fan, S., Liu, K., Cao, Z. & Wang, D. (2010). *Acta Cryst.* E**66**, m77. 10.1107/S1600536809053720 PMC2980178 21579970

[bb22] Refat, M. S., El-Korashy, S. A., Kumar, D. N. & Ahmed, A. S. (2008). *Spectrochim. Acta Part A*, **70**, 898–906. 10.1016/j.saa.2007.10.005 18024192

[bb23] Shahverdizadeh, G. H. & Tiekink, E. R. T. (2011). *Acta Cryst.* E**67**, o798. 10.1107/S1600536811007847 PMC3100046 21754087

[bb24] Sheldrick, G. M. (2008). *Acta Cryst.* A**64**, 112–122. 10.1107/S0108767307043930 18156677

[bb25] Sheldrick, G. M. (2015*a*). *Acta Cryst.* A**71**, 3–8.

[bb26] Sheldrick, G. M. (2015*b*). *Acta Cryst.* C**71**, 3–8.

[bb27] Soroceanu, A., Shova, S., Cazacu, M., Balan, I., Gorinchoy, N. & Turta, C. (2013). *J. Chem. Crystallogr.* **43**, 310–318.

[bb28] Spackman, M. A. & Jayatilaka, D. (2009). *CrystEngComm*, **11**, 19–32.

[bb29] Spek, A. L. (2009). *Acta Cryst.* D**65**, 148–155. 10.1107/S090744490804362X PMC2631630 19171970

[bb30] Turner, M. J., McKinnon, J. J., Wolff, S. K., Grimwood, D. J., Spackman, P. R., Jayatilaka, D. & Spackman, M. A. (2017). *CrystalExplorer17. University of Western Australia.* http://hirshfeldsurface.net

[bb31] Yoshida, N. & Ichikawa, K. (1997). *Chem. Commun.* pp. 1091–1092.

